# Subtle modulation of ongoing calcium dynamics in astrocytic microdomains by sensory inputs

**DOI:** 10.14814/phy2.12454

**Published:** 2015-10-05

**Authors:** Akiko Asada, Sakiko Ujita, Ryota Nakayama, Shigeyuki Oba, Shin Ishii, Norio Matsuki, Yuji Ikegaya

**Affiliations:** 1 Graduate School of Pharmaceutical Sciences, University of TokyoTokyo, Japan; 2 Graduate School of Informatics, Kyoto UniversityKyoto, Japan; 3 Center for Information and Neural NetworksSuita City, Osaka, Japan

**Keywords:** Astrocyte, endfoot, map, orientation selectivity, visual cortex

## Abstract

Astrocytes communicate with neurons through their processes. In vitro experiments have demonstrated that astrocytic processes exhibit calcium activity both spontaneously and in response to external stimuli; however, it has not been fully determined whether and how astrocytic subcellular domains respond to sensory input in vivo. We visualized the calcium signals in astrocytes in the primary visual cortex of awake, head-fixed mice. Bias-free analyses of two-photon imaging data revealed that calcium activity prevailed in astrocytic subcellular domains, was coordinated with variable spot-like patterns, and was dominantly spontaneous. Indeed, visual stimuli did not affect the frequency of calcium domain activity, but it increased the domain size, whereas tetrodotoxin reduced the sizes of spontaneous calcium domains and abolished their visual responses. The “evoked” domain activity exhibited no apparent orientation tuning and was distributed unevenly within the cell, constituting multiple active hotspots that were often also recruited in spontaneous activity. The hotspots existed dominantly in the somata and endfeet of astrocytes. Thus, the patterns of astrocytic calcium dynamics are intrinsically constrained and are subject to minor but significant modulation by sensory input.

## Introduction

Astrocytes, a predominant type of glial cells in the brain, extend their processes and make contacts with enormous numbers of synapses and blood vessels (Mulligan and MacVicar 2004; Halassa et al. 2007). Through those contacts, astrocytes regulate the maintenance and development of the nervous system (Nishida and Okabe 2007; Pellerin et al. 2007). Previous studies have shown that astrocytes respond to intense neuronal activity with intracellular calcium elevations (Porter and McCarthy 1996; Panatier et al. 2011). Calcium activity may modulate the release of gliotransmitters and thereby modulate synaptic transmission and plasticity (Perea et al. 2009; Henneberger et al. 2010). However, such knowledge has been accumulated primarily through in vitro preparations, and the extent of neuron-to-glial interactions under more physiological conditions remains controversial (Fiacco et al. 2007; Agulhon et al. 2010; Sun et al. 2014).

**Figure 1:**
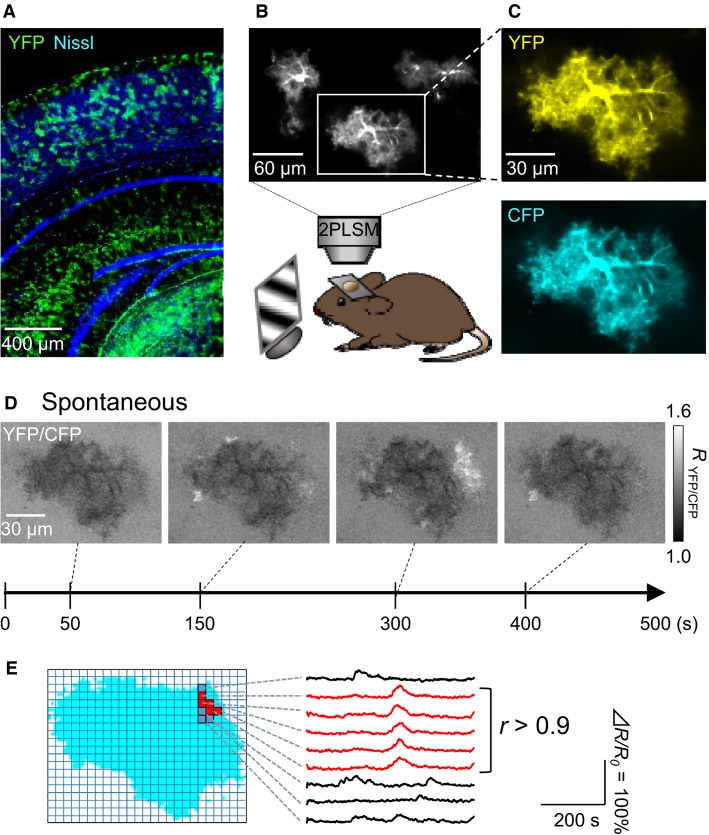
Subcellular calcium activity in astrocytes of awake mice. (A), Nissl-stained coronal section of an *Mlc1*-tTA::tetO-YC-Nano50 mouse. (B), Two-photon image of three YC-Nano50-expressing astrocytes in layer 2/3 of the primary visual cortex. (C) YFP (top) and CFP (bottom) fluorescence images of the astrocyte boxed in B. (D), Representative time-lapse images of the YFP/CFP ratio (*R*) of the astrocyte shown in C. (E), Schematic diagram for the detection of subcellular calcium domains. A contiguous aggregate of adjacent pixels with correlation coefficients (*r*) higher than 0.9 was defined as a “domain” (red).

In vivo studies have demonstrated that whisker deflections, hind-limb shocks, and air puffs trigger synchronized calcium signals in multiple astrocytes (Wang et al. 2006; Winship et al. 2007; Schummers et al. 2008; Takata et al. 2011; Navarrete et al. 2012; Ding et al. 2013; Paukert et al. 2014), which may represent startle responses to robust or noxious stimuli. By contrast, milder and noninvasive sensory stimuli, such as visual input, have failed to reliably activate astrocytes. However, these studies focused mainly on calcium activity in the cell bodies. Because astrocytic processes often generate calcium activity that is independent of the somatic activity (Volterra et al. 2014), the sensory responses of subcellular domains of astrocytes, such as processes and endfeet, remain to be examined. In the present work, we used *Mlc1*-tTA::tetO-YC-Nano50 transgenic mice (Kanemaru et al. 2014), which sparsely express yellow cameleon-Nano50 (YC-Nano50), a high-sensitivity genetically encoded calcium indicator, in a small fraction of neocortical astrocytes (Fig.1A), to image calcium activities from astrocytic processes.

Moreover, conventional methods to detect and analyze calcium signals have usually included manual or arbitrary settings to define the regions of interest. These human-biased methods require some preknowledge about patterns in calcium dynamics and may be subject to false-positive or false-negative errors in detection of calcium activity if the true activity is spatiotemporally unstructured. Therefore, we invented a novel algorithm to unbiasedly detect astrocytic calcium activity, which allowed us to analyze the spatiotemporal information of calcium activity.

During observation of calcium activity of astrocytes from the primary visual cortex, we presented visual stimuli to mouse eyes. We then examined the difference between spontaneous and evoked calcium activity and statistically evaluated the spatial biases of these responses within individual astrocytes. We found that astrocytes spontaneously exhibited spot-like calcium signals that were inhomogenously distributed over the entire cell. In addition, visual stimulation did not strongly change these spatial activity patterns, although it modulated the sizes of ongoing calcium domains.

## Methods

### Ethical approval

All experiments were performed with the approval of the Animal Experimental Ethics Committee at the University of Tokyo (approval number: P21-6) and according to the University of Tokyo guidelines for the care and use of laboratory animals.

### Surgery

Male *Mlc1*-tTA::tetO-YC-Nano50 transgenic mice aged 30–55 day were anesthetized with ketamine (50 mg/kg, i.p.) and xylazine (10 mg/kg, i.p.) and were implanted with a metal head-holding plate. They were subjected to head-fixation training for 5–10 day, as described previously (Ishikawa et al. 2014). The mice were again anesthetized with a ketamine/xylazine cocktail, and a small craniotomy (1 × 1 mm^2^) was made over the primary visual cortex at 3.5 mm caudal to the bregma and 2 mm ventrolateral to the sagittal suture. The exposed cortical surface was covered with 2% agar. After the mice recovered from anesthesia, recordings were made under head fixation. In some experiments, animals were ventilated with a volume cycled ventilator at a rate of 110 breaths/min with 9 mL/kg while paralysis was introduced with pancuronium bromide (0.3 mg/kg, i.p.) to minimize muscle movements (Minamisawa et al. 2011). Data obtained from the awake and acutely paralyzed mice were pooled in the following analyses, because similar results were obtained under these experimental conditions, indicating that our results were not subject to an artifact due to body motion or eye blink. Throughout the experiment, a heating pad maintained the rectal temperature at 37°C.

### Calcium imaging

Calcium activity was imaged at 1 Hz using an FV1000 two-photon laser-scanning microscope (Olympus) (Fig.1B). Fluorophores were excited at 900 nm, and fluorescence images were collected through a water-immersion 25× objective lens (1.05 NA). CFP and YFP signals were divided by a dichroic mirror (505 nm) and 460-to-510-nm and 510-to-560-nm band-path filters (Fig.1C), respectively, and were recorded using two photomultipliers. The signal intensity ratio of YFP to CFP was calculated as the calcium signal. The microscopic field of view covered approximately 350 × 200 *μ*m^2^ and contained 4.0 ± 0.6 YC-Nano50(+) astrocytes (mean ± SD of 15 imaging fields).

### Blood vessel imaging

Blood vessels were imaged via intravenous injection of 50–100 *μ*L of 0.3% fluorescein isothiocyanate (FITC)-labeled dextran (70 kDa) into the tail vein. Fluorophores were excited at 800 nm and fluorescence images were collected through the objective lens.

### Visual stimuli

**Figure 2:**
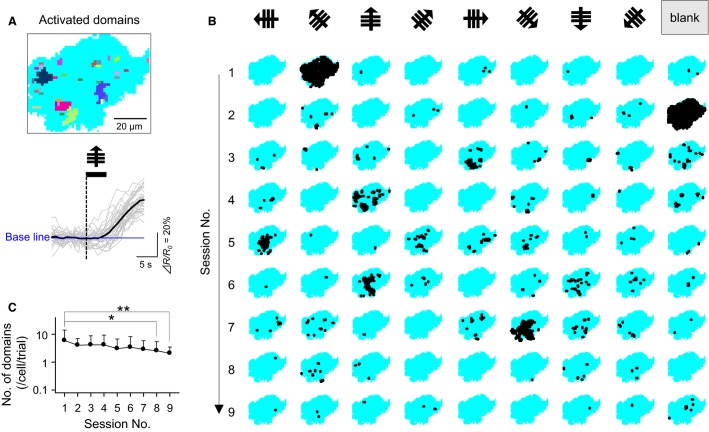
Visual responses of calcium signals in single astrocytes. (A), Representative map of 31 activated domains in a single astrocyte (top). Different colors indicate individual domains. The raw traces of their fluorescence intensities are superimposed on the thick-lined average trace (bottom). The dashed line indicates the onset time of an upward-drifting grating stimulus. (B), Activated domains observed in a single astrocyte during nine sessions, each of which contained gratings with eight directions (arrows) and a gray screen (blank). Black areas indicate domains. (C), The average number of activated domains in a single cell per trial as a function of the sessions. Error bars indicate the SDs of 36 cells from nine videos. *P =* *0.037, **P = 0.008, Scheffe’s test.

Drifting grating stimuli (100% contrast; temporal frequency: 2 Hz; spatial frequency: 0.04–0.05 cycles per degree) with eight motion directions at every 45° step were presented for 5 sec on a 17-inch monitor (refresh rate: 60 Hz; max luminance: 240 cd/m^2^), which was located 30 cm from the eye contralateral to the imaged hemisphere. A gray-blank screen (120 cd/m^2^) was displayed during the inter-stimulus intervals, which lasted 55 sec. In each session, gratings with eight directions and a gray blank (120 cd/m^2^) were sequentially presented every 60 sec in a pseudorandom order. The blank was used to record spontaneous activity (Fig.2B). The session was cycled nine times.

### Drug application

The cortical surface was perfused with 100 *μ*mol/L tetrodotoxin for 10 min and covered with 2% agar containing 2 *μ*mol/L tetrodotoxin.

### Data analysis

The data were analyzed using custom-made MATLAB software (Math Works; Natick, MA). The cell contours were determined based on the YFP images. Each image was dissected into lattices of 2 × 2 imaged pixels, which were re-defined herein as ‘pixels,’ corresponding to approximately 1.5 × 1.5 *μ*m^2^. For a given 25-sec period in a time series of the YFP-to-CFP ratio (*R*), we measured the correlation coefficients (*r*) between two contiguous pixels and extracted a ‘domain’ in which all *r* values were greater than 0.9 (Fig.1E). To determine whether a domain was visually evoked, we focused on the 25-sec time window that consisted of the 10 sec before the stimulus and the 15 sec after the stimulus (Fig.2A bottom). In the domain, Δ*R/R*_0_ was calculated as (*R*_*t*_* *− *R*_0_)/*R*_0_, where *R*_*t*_ is the fluorescence change at time *t* and *R*_0_ is the baseline value averaged over the 5-sec baseline period prior to the visual stimulus onset. Changes were considered responses to visual stimuli when Δ*R/R*_0_ exceeded 2 × SDs of the baseline for >1 sec within 15 sec after the onset of the stimulus. The domain with visual responses was defined as an ‘activated domain’ (Fig.2B). Importantly, the event frequencies in the activated domains were reduced over nine sessions (Fig.2C; 1^st^ session vs. 8^th^ session: *P *=* *0.037, MD = 2.1, 1^st^ session vs. 9^th^ session: *P *=* *0.008, MD = 2.3; Scheffe’s test after one-way ANOVA, *n *= 36 cells in nine videos from nine mice), suggesting that repetitive stimuli altered the calcium dynamics, likely because of sensory adaptation or phototoxicity. To exclude such artifacts, we used the first six sessions in the analyses. Moreover, trials in which calcium activity at the whole-cell level (>10% of the cell area) occurred simultaneously in at least two cells were excluded from the subsequent analyses because such large synchronizations more likely to reflect locomotion and startle responses rather than visual responses (Ding et al. 2013; Paukert et al. 2014). As a result, approximately 5% of the trials were excluded from the analyses. In Figure4, the orientation selectivity index (OSI) and the direction selectivity index (DSI) were defined as follows: 

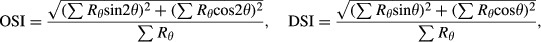
where *θ* represents the direction of the grating stimuli, and *R*_*θ*_ is the total number of activated domains (Fig. 4B), the total area of activated domains (Fig. 4C), the mean size of individual activated domains (Fig. 4D), or the response rate of pixels that responded at least twice to a single direction (Fig. 5C). To evaluate the response reliability, the *Z* score was defined as follows: 

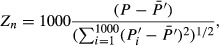
where *P* represents the response rate of pixel *n*, and 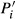 and 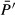 represent the response rate of the *i*^th^ dataset of a total of 1000 surrogates for pixel *n* and their mean across the 1000 surrogates, respectively. To quantify the spatial bias of *Z*, the geometric energies were calculated as follows (Makino et al. 2015): 

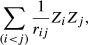


where *Z*_*i*_ and *Z*_*j*_ are the *Z* scores of pixels *i* and *j*, respectively, and *r*_*ij*_ is the Euclidean distance between pixels *i* and *j*.

### Statistics

All data are expressed as means ± SDs unless otherwise specified. Student’s *t*-test, paired *t*-test, Welch’s *t*-test and one-way ANOVA were used to test the statistical significance of the mean data. Tukey’s test and Scheffe’s test were used for multiple comparisons. The Kolmogorov–Smirnoff (KS) test was used to compare the cumulative probability distributions. Product-moment correlation coefficient test was used to determine whether the correlations reached the significance level. Significance was considered for *P *< 0.05.

## Results

### Visual responses of astrocytic calcium domains

We monitored subcellular calcium activity from a total of 60 astrocytes in layer 2/3 of the monocular primary visual cortex in 15 videos that were taken in 15 *Mlc1*-tTA::tetO-YC-Nano50 mice (Fig.1; *n *= 39 cells from 10 control mice and 21 cells from 5 tetrodotoxin-treated mice). During our 90-min observation period, all 60 astrocytes exhibited spontaneous calcium elevations. Most of the calcium activity events did not recruit the entire cell but were spatially restricted to small regions within the astrocytes. Because the spatial patterns of the activity were likely unstructured, we developed an unbiased method to define calcium-responsive “domains” based on the spatiotemporal correlations of calcium fluorescence (Fig.1E, see the Methods section). In single astrocytes, on average, calcium domains appeared spontaneously at event frequencies of 3.4 ± 0.5 per 15 sec (mean ± SEM of 39 astrocytes in 10 control mice). A single domain averaged 7.4 ± 0.9 *μ*m^2^, which corresponded to approximately 0.2% of the whole-cell area (mean ± SEM of 720 domains; ranging from 3.5 to 241 *μ*m^2^).

**Figure 3:**
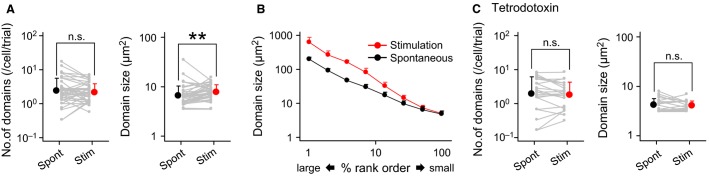
Visual stimulation induces larger calcium domains. (A), The mean number of calcium domains in a single cell per trial (left) and the mean size of individual domains (right) are compared between spontaneously emerging domains (Spont) and visually activated domains (Stim). Error bars are the SDs of 39 cells. Each gray line indicates a single astrocyte. **P =* *0.009, paired *t*-test. (B), Percentage rank orders of the sizes of 5236 (Stimulation) and 720 (Spontaneous) domains. (C), Same as A, but for tetrodotoxin-treated visual cortex. *n *= 21 astrocytes.

To examine whether astrocytic calcium domains respond to visual stimuli, we presented conventional gray-scale grating stimuli that drifted with eight directions to one eye of each mouse and recorded astrocytic calcium activity from the contralateral visual cortex. Calcium domains whose occurrence times were locked to the visual stimulation onset are described here as ‘activated domains’ (Fig.2A, see the Methods section). Activated domains were sparse over the trials and even varied in their responses to identical directional stimuli (Fig.2B), indicating that the visual responses of the astrocytes were not stable in terms of response probability or location. Importantly, the event frequencies of the activated domains did not differ from those of spontaneously emerging domains (Fig.3A left; *P *= 0.29, *t*_38_ = 1.1, paired *t-*test, *n *=* *5236 activated and 720 spontaneous domains in 39 cells from 10 mice); that is, visual stimuli did not increase the number of calcium domains beyond the background activity frequency. Nonetheless, the activated domains exhibited slightly but significantly larger sizes compared to spontaneous domains (Fig.3A right; *P *= 0.009, *t*_38_ = 2.7, paired *t-*test). Further analyses revealed that visual stimuli preferentially enlarged larger domains, whereas they exerted less of an effect on smaller domains (Fig.3B).

To examine whether the visual modulation of astrocytic calcium domains was triggered by neuronal spiking activity, we applied tetrodotoxin, an inhibitor of voltage-sensitive sodium channels, on the surface of the visual cortex. Under blockade of neuronal spiking activity, the event frequency of spontaneous domains was similar to that of the control mice (Fig.3C left; *P *= 0.44, *t*_58_ = 0.77, Student’s *t*-test), but the mean domain size was significantly smaller than in the controls (Fig.3C right; *P *= 3.1 × 10^−5^, *t*_58_ = 4.5, Welch’s *t*-test). Thus, astrocytic domains emerged independent of neuronal spiking activity, but their sizes were modulated by ongoing neuronal activity. Consistent with this idea, the visually evoked enlargement of calcium domains did not occur in the presence of tetrodotoxin (Fig.3C; number: *P *= 0.52, *t*_20_ = 0.66; area: *P *= 0.50, *t*_20_ = 0.69; paired *t-*test).

### Nonsignificant response tuning

**Figure 4:**
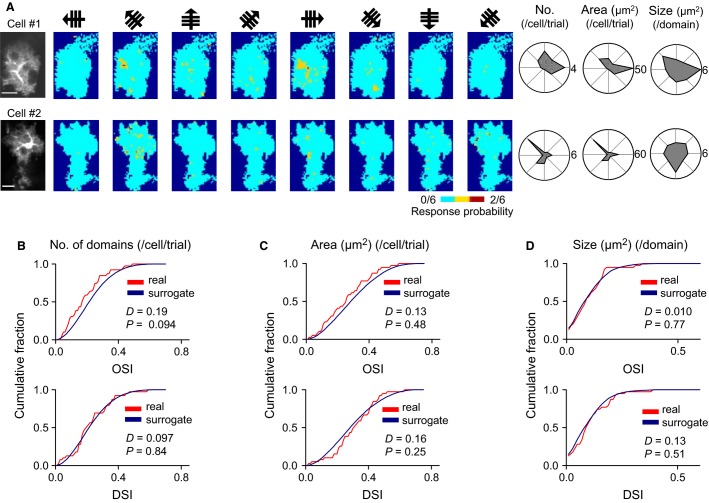
Nonsignificant orientation tuning of astrocytic calcium signals at the whole-cell level. (A), Maps of activated domains stacked over six sessions for each direction in two representative astrocytes (left). Scale bar=20 *μ*m. The right polar plots for these two astrocytes indicate the number of activated domains per trial (No.), the total area of activated domains per trial (Area), and the mean size of individual domains (Size) for gratings with eight directions. (B–D), The cumulative distributions of OSIs and DSIs for the number of activated domains (B), the total area of activated domains (C), and the mean size of individual activated domains (D) were compared between real datasets (red) and their 1000 trial-shuffled surrogates (blue). The *D* and *P* values were determined using the Kolmogorov–Smirnov test. *n *= 39 astrocytes.

Mouse visual neurons show response selectivity with preferred orientations and directions of visual stimuli (Niell and Stryker 2008). We, thus, investigated the response tuning of astrocytic calcium domains. For each astrocyte, we collected all of the activated domains in six sessions (i.e., 48 trials) and plotted their occurrence probability for the individual grating directions (Fig.4A left). For each direction, we calculated three parameters, including the total number of domains, the summed area of domains, and the mean size of individual domains, and these data are shown in polar coordinates with the eight directions (Fig.4A right). To quantify the direction bias in the plots (i.e., response preference), we computed OSIs and DSIs. The OSIs and DSIs are pooled from 39 cells in 10 mice, and their distributions are shown for the three parameters (Figs.4B–D). For statistical analysis, we compared these calculations to their chance distributions obtained from 1000 surrogate datasets in which the activated domains were randomly shuffled across trials. None of the parameters had significantly increased OSIs or DSIs (Kolmogorov–Smirnov test), suggesting that the activated domains have no apparent response preferences, at least at the whole-cell level.

**Figure 5:**
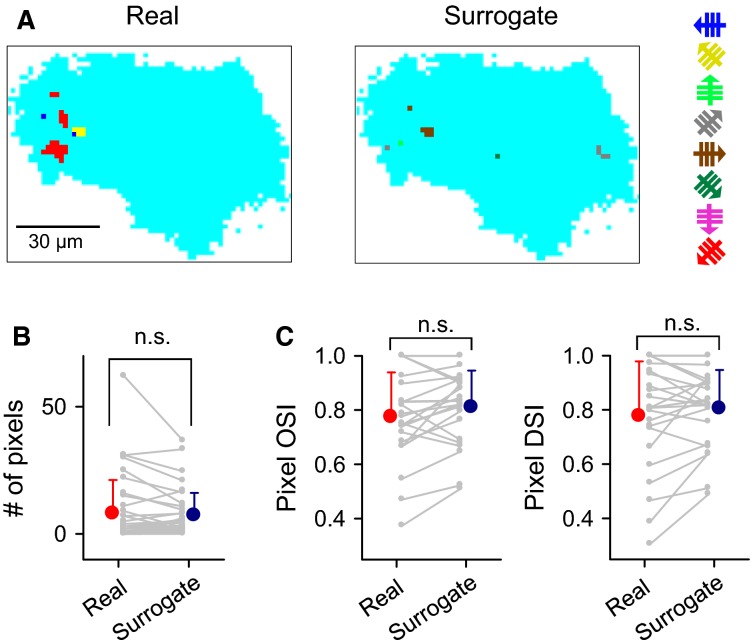
Nonsignificant orientation tuning of astrocytic calcium signals at the calcium domain level. (A), Map of pixels that responded at least twice to a single direction in real datasets (left) and 1000 trial-shuffled surrogates (right). Colors indicate the preferred directions to which each pixel responded most frequently. (B), Mean numbers of responsive pixels in 37 real datasets and their pixel-shuffled surrogates. Gray lines indicate single astrocytes, and error bars represent the SD. (C), Mean ± SD of OSIs (left) and DSIs (right) of the responsive pixels in 23 real datasets and their surrogate data.

To examine the response tuning at the subcellular level, we next analyzed the OSIs and DSIs for individual pixels. As reliable ‘responsive’ pixels, we selected pixels that were activated by stimuli with the identical direction in at least two of the six sessions (Fig.5A). The responsive pixels accounted for 0.5 ± 0.1% of the total pixels in single cells (mean ± SD of 37 cells from 10 mice). The total number of responsive pixels per cell did not differ from the chance level as estimated based on 1000 surrogates in which individual pixels comprising activated domains were randomly shuffled within each trial (Fig.5B; *P = *0.084, *t*_36_ = 1.8, paired *t-*test). Moreover, neither the OSIs nor the DSIs of individual pixels were significantly higher than the chance level in the surrogates (Fig.5C; OSI: *P = *0.059, *t*_22_ = 2.0; DSI: *P = *0.21, *t*_22_ = 1.3; paired *t-*test). Thus, individual pixels did not respond preferentially to a specific grating direction.

### Hotspots of calcium domains

**Figure 6:**
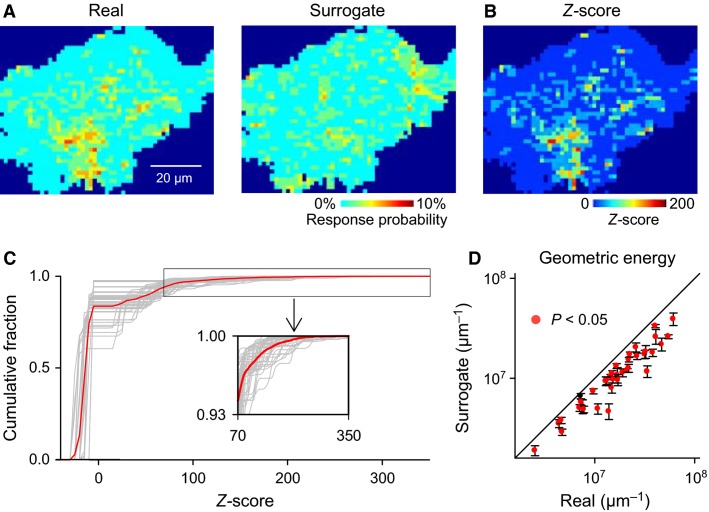
Hotspots for astrocytic calcium signals. (A), Heat maps of activated domains stacked across all 48 trials during six sessions in real data (left) and domain-shuffled surrogates (right). (B), The *Z* scores calculated from the response probabilities of real and surrogate datasets in A are shown for each pixel in a pseudocolored scale. (C), The cumulative distribution of the *Z* scores of individual pixels in each astrocyte (gray) is superimposed on their average (red), indicating that a few pixels exhibited extremely high-*Z* values. (D), The mean geometric energy of the spatial distribution of the *Z* score within individual cells is compared between 37 real datasets and their 1,000 domain-shuffled surrogates. Red dots represent astrocytes with significantly high-geometric energy, indicating that highly responsive pixels were spatially clustered.

Previous studies have suggested that astrocytes exhibit spatially biased subcellular calcium activity (Shigetomi et al. 2013). To assess the existence of subcellular regions that were responsive to visual stimuli, we plotted a responsive-area map without reference to the stimulation direction. The response probability, the ratio of trials with calcium activation to a total of 48 trials (8 directions × 6 sessions), is shown for every pixel within an astrocyte using a pseudocolored scale (Fig.6A left). We repeated the same analysis for 1000 surrogates in which the activated domains were randomly scattered within each trial (Fig.6A right). Based on the chance distribution estimated by the surrogates, we calculated the *Z* score of each pixel (Fig.6B). This *Z*-score map suggests that some pixels were highly responsive. Indeed, the distribution of the *Z* scores within the individual cells was skewed by a few select pixels with extremely high-*Z* scores (Fig.6C), resulting in a mean Gini coefficient of 0.96 ± 0.01 (mean ± SD of 37 cells).

To further examine the spatial bias of the *Z* scores, we introduced the geometric energies, which shows a higher value for a more spatially clustered distribution. In 36 of 37 cells, the geometric energies of the real datasets were significantly higher (*P *< 0.05) than the average of 1000 domain-scattered surrogates (Fig.6D), indicating that highly responsive pixels were localized in astrocyte subregions (“hotspots”).

**Figure 7:**
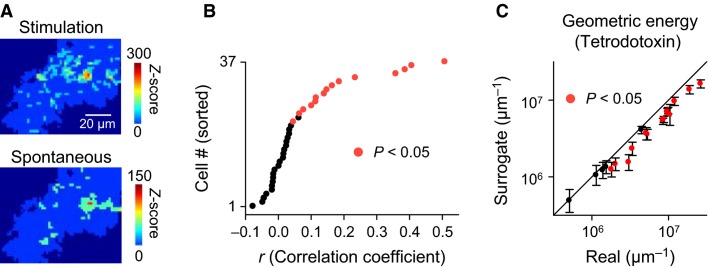
Visual responses resemble spontaneous activity patterns. (A), Representative *Z*-score maps in activated domains (Stimulation) and spontaneous domains (Spontaneous). (B), Spatial correlations of the *Z* scores in all pixels between Stimulation and Spontaneous conditions. Red dots indicate astrocytes with significantly high correlation coefficients (Pearson product–moment correlation coefficient test). (C), Same as Fig.6D, but for spontaneous domains that occurred in tetrodotoxin-treated visual cortex. *n *= 19 astrocytes.

We next computed the *Z*-score maps for spontaneously occurring domains. Spontaneous domains also had hotspot-like spatial biases (Fig.7A). For each cell, we calculated the spatial correlation coefficients of the *Z* scores for activated and spontaneous domains. In 15 of 37 astrocytes, the correlations reached the significance level of *P *< 0.05 (Fig.7B; Pearson product-moment correlation coefficient test), indicating that the spatial patterns of the *Z*-score maps were similar between activated and spontaneous domains. Interestingly, the geometric energies of the *Z*-score maps of spontaneous domains in the tetrodotoxin-treated cortex were still significantly higher than their surrogates in 14 of 19 astrocytes (Fig.7C, *n* = 5 mice), suggesting that hotspots emerge cell-autonomously.

**Figure 8:**
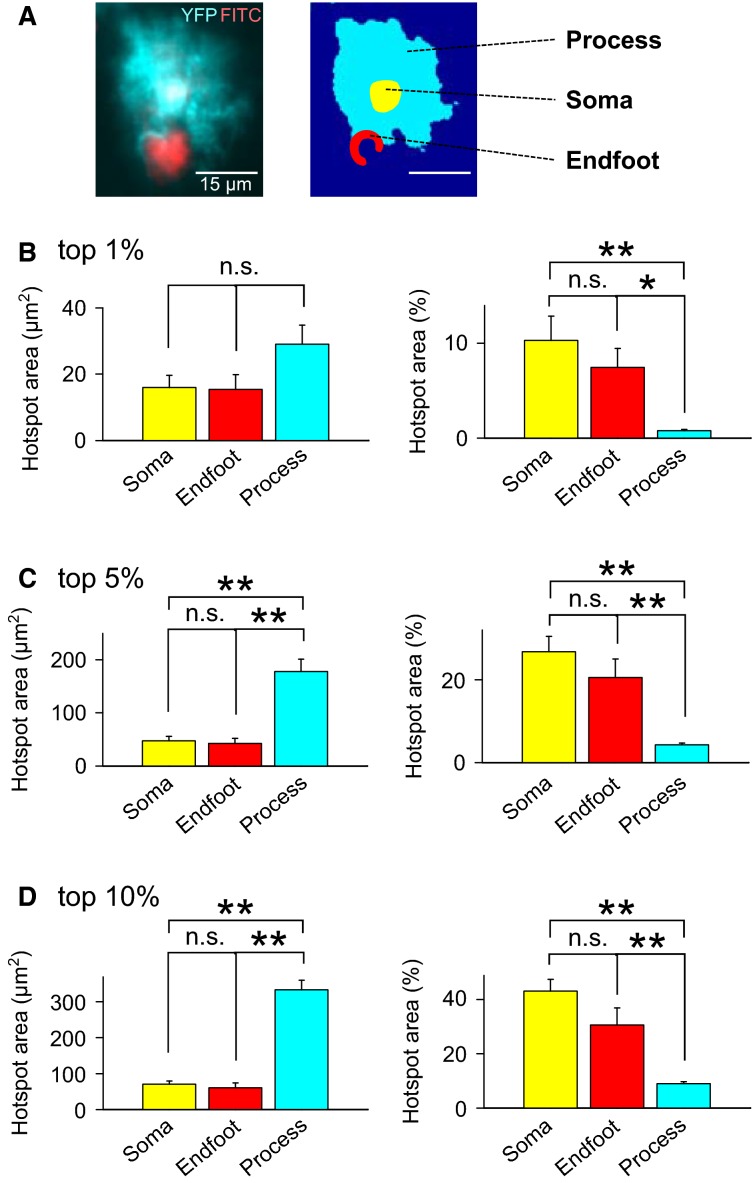
Subcellular localization of calcium hotspots. (A), Representative two-photon image of an astrocyte (YFP, cyan) and FITC-dextran-injected blood vessels (FITC, red) in the layer 2/3 of the primary visual cortex (left). The cell was divided into three regions, soma, process, and endfoot (right). (B–D), Pixels with *Z* scores in the top 1% (B), 5% (C), and 10% (D) in their entire distributions were defined as “hotpixels”. The total areas (left) and the ratios (right) of hotpixels to the total area in the three zones are plotted. Error bars are SEMs of 33 cells (soma), 22 cells (endfoot), and 38 cells (process) from 10 videos. *P <* *0.05, **P < 0.01, Tukey’s test after one-way ANOVA.

An astrocyte has three different subcellular structures, the soma, processes, and endfoot. The endfoot is a specialized zone that surrounds a brain blood vessel. By visualizing vascular networks through intravenous injection of FITC-dextran, we divided astrocytes into these three subregions (Fig.8A). To quantify the distribution of hotspots across these subregions, we defined pixels with the top 1% of the *Z* scores in all imaged pixels as “hotpixels”. The total areas of hotpixels did not differ among three subregions (Fig.8B left; *P *= 0.087; *F*_2_,_90_ = 2.5; one way ANOVA, *n *= 33 cells (soma), 22 cells (endfoot) and 38 cells (process) in 10 videos from 10 mice). On the other hand, the ratios of hotpixels to the total areas of subregions were significantly higher in the somata and endfeet than in processes (Fig.8B right; soma vs. process, *P *=* *0.0004, *Q*_2_,_90_ = 5.6; endfoot *versus* process *P *=* *0.039, *Q*_2_,_90_ = 3.5; Tukey’s test after one way ANOVA), indicating that hotspots were more concentrated in the somata and endfeet, compared to processes. Consistent with this result, hotpixels with the top 5% and the top 10% of the *Z* scores exhibited higher ratios in the somata and endfeet of astrocytes (Fig.8C and D; **P < 0.01; Tukey’s test after one way ANOVA).

## Discussion

In this study, we monitored subcellular calcium activity using transgenic mice that express an ultrasensitive calcium indicator in a fraction of astrocytes, which allowed us to record the calcium dynamics in astrocytic compartments without signal contamination from adjacent astrocytes. Moreover, bias-free data analyses allowed us to discover the diverse spatial-temporal patterns of astrocytic calcium microdomains. Astrocytes spontaneously exhibited spot-like calcium activity in their subcellular regions; the somata and endfeet frequently emitted such localized calcium activity and formed calcium hotspots. The hotspots were likely fixed independently of neuronal spiking activity. However, tetrodotoxin treatment shrank the sizes of the calcium domains, whereas visual stimuli enlarged them. Because neither tetrodotoxin nor visual stimuli affected the event frequency of calcium domains, neuronal activity is unlikely to regulate the incidence of calcium domains but is more likely to modulate the sizes of spontaneously emerging calcium domains. Consistent with this idea, the locations of hotspots of calcium domains were not altered by visual stimuli, nor did the calcium domains possess orientation preferences during visual stimuli. Therefore, we conclude that visual stimulus-locked astrocytic activity represents a small modulation of ongoing calcium dynamics by the input signal, rather than reflecting the structure of the input signal itself.

The visually evoked enlargement of calcium domains is consistent with a recent in vitro study showing that stimulation of axonal fibers increases the sizes, but not the frequencies, of subcellular calcium events (Wu et al. 2014). The calcium domains are expected to increase in three dimensions, but in these studies, including ours, they were measured in two dimensions. Thus, the modulation of the domain sizes may be underestimated. This could be particularly the case for smaller domains, and indeed, we observed the sensory modulation in only large calcium domains. Anatomical investigation has demonstrated that the processes of a single astrocyte cover a volume of 15,000 *μ*m^3^ (Chvatal et al. 2007), and they may make contact with >50,000 synapses (Rusakov et al. 1999). Therefore, a calcium domain with a diameter of 10 *μ*m is expected to contain >200 synapses. Based on our data, these synapse populations are unlikely to trigger calcium responses in astrocytic processes, but they may intensify endogenous calcium dynamics when their activity coincides with astrocytic calcium activity. Astrocytic processes detect the activity of glutamatergic synapses via their metabotropic glutamate receptors and ionotropic glutamate receptors (Palygin et al. 2010; Panatier et al. 2011; Rusakov et al. 2011). These receptors are coupled with intracellular calcium signaling. Of note is the observation that activation of metabotropic glutamate receptors changes the sizes (but not frequencies) of calcium signals (Wu et al. 2014). Therefore, they may mediate an interaction between synaptic activity and astrocytic calcium domains. To identify the intercellular signal underlying the modulation of calcium domains, it is essential to conduct pharmacological or genetic manipulations that specifically block astrocytic receptors. A recent in vivo work has shown that small activities in astrocytic processes are more frequent than whole-cell level activities (Kanemaru et al. 2014). Another in vitro study has showed that these small signals of astrocytes modulate basal transmission at synapses that contact with the active astrocytic process (Rusakov et al. 2014; Volterra et al. 2014). Thus, we believe that the calcium microdomains observed in our study are physiologically relevant; that is, sensory input-induced enlargement of the microdomains may alter neurotransmissions at the surrounding synapses.

Calcium domains were coordinated into discrete hotspots and emerged unevenly within the astrocytes. The hotspots were preferentially localized in somata and endfeet, although they were numerically dominant in processes (because processes contributed a larger volume proportion of an astrocyte). Astrocytes possess intracellular calcium stores, including the endoplasmic reticulum and mitochondria, both of which can generate intracellular calcium releases (Parpura et al. 1994; Nett et al. 2002). These subcellular organelles are localized heterogeneously across astrocytic subcellular regions; peri-synaptic processes are often devoid of calcium stores, whereas the stores are more abundant in thicker structures (Panatier et al. 2011; Jackson et al. 2014). These subcellular biases are seemingly consistent with our findings that hotspots were concentrated in large structures such as endfeet and somata, but not in processes. Previous studies have shown that astrocytic calcium activity contributes to extracellular ionic concentrations and thereby the transformation of astrocytic structures, which may in turn change the synaptic contact ratio and the diffusion efficiency of neurotransmitters (Lippman Bell et al. 2010; Wang et al. 2012; Tanaka et al. 2013). Thus, calcium hotspots may have a role in the homeostatic feedback along synaptic activity levels. Moreover, because astrocytic calcium activity can modify cerebral vascular microcirculation (Petzold and Murthy 2011) the hotspots in endfoot may regulate local energy supplies via local blood flow.

In conclusion, the calcium dynamics of astrocytes were spatiotemporally sparse, predominantly spontaneous, and minimally subject to sensory modulation. It should be noted these findings are in marked contrast with previous reports showing that artificial stimulation reliably evokes calcium responses in astrocytes. To our knowledge, our work is the first to focus on the responses of astrocytic subcellular components to mild sensory stimuli. To reduce false-positive detection of noise signal due to in vivo imaging, we adopted a threshold for detection of calcium activity and discarded very small activity with the area sizes of less than 1.5 × 1.5 *μ*m^2^. However, an in vitro study has suggested that such tiny calcium events occur in astrocytic processes (Di Castro et al. 2011). Further investigations using natural stimulation in vivo, manipulations of calcium domains, and better signal detection methods will help resolve the recent controversy regarding the function of astrocytic calcium activity (Bernardinelli et al. 2014; Rusakov et al. 2014; Volterra et al. 2014).
